# A Comparison of Regulatory Maternity Unit Ratings With Clinical Outcomes and Practice Measures: An Observational Study Using Routinely Collected Data

**DOI:** 10.1111/1471-0528.18188

**Published:** 2025-05-05

**Authors:** Ian Henderson, Ipek Gurol‐Urganci, Alissa Frémeaux, Alessandra Morelli, Kirstin Webster, Amar M. Karia, Fran Carroll, George Dunn, James Harris, Sam Oddie, Asma Khalil, Jan van der Meulen

**Affiliations:** ^1^ Department of Health Services Research & Policy London School of Hygiene & Tropical Medicine London UK; ^2^ Centre for Quality Improvement and Clinical Audit Royal College of Obstetricians and Gynaecologists London UK; ^3^ Chelsea and Westminster Hospital NHS Foundation Trust London UK; ^4^ Bradford Institute for Health Research Bradford Royal Infirmary Bradford UK; ^5^ Fetal Medicine Unit, Department of Obstetrics and Gynaecology St George's University Hospitals NHS Foundation Trust London UK; ^6^ Vascular Biology Research Centre Molecular and Clinical Sciences Research Institute, City St George's University of London London UK

**Keywords:** caesarean, induction, morbidity, pregnancy, safety

## Abstract

**Objective:**

To compare inspection‐informed ratings of individual maternity units published by the Care Quality Commission (CQC) with clinical outcomes and practice measures.

**Design:**

Observational study using linked national maternity and administrative hospital data.

**Setting:**

The English NHS.

**Population:**

Women with singleton pregnancies who gave birth at term, April 2018–March 2019.

**Methods:**

Outcomes and practice measures were compared with ratings using hierarchical models and empirical Bayes estimates adjusted for case‐mix and unit characteristics.

**Main Outcome Measures:**

Severe maternal and severe neonatal morbidity. Practice measures included non‐spontaneous birth (either caesarean birth before labour or the induction of labour) and intrapartum caesarean birth.

**Results:**

Of 501 719 included women, 39 930 (8.0%) gave birth in 11 units rated ‘outstanding’, 357 114 (71.2%) in 110 units rated ‘good’, and 104 675 (20.9%) in 35 units rated ‘requires improvement/inadequate’. Severe maternal morbidity did not vary by rating: 1.2% [95% confidence interval 0.87–1.5], 1.3% [1.1–1.4], and 1.0% [0.87–1.1], respectively (*p* = 0.59), nor did the risk of severe neonatal morbidity: 4.3% [3.3–5.6], 4.0% [3.6–4.5], and 3.4% [2.9–3.9], respectively (*p* = 0.48). There was no variation across the ratings in the rate of non‐spontaneous birth (48.1% [42.2–53.9], 47.9% [46.4–49.4], and 47.9% [45.1–50.8], respectively; *p* = 0.87) nor intrapartum caesarean (16.8% [14.6–19.3], 16.6% [15.8–17.3], and 15.8% [14.9–16.7], respectively; *p* = 0.87).

**Conclusions:**

There was no association between ratings of maternity units published by the national healthcare regulator and clinical outcomes and practice measures derived from routinely collected data. Concerted action is urgently needed to improve the inspection‐informed ratings of maternity services.

## 1 Introduction

Maternity care is intensely monitored in many high‐income countries. In England, there are perinatal mortality surveillance [1] and review systems [2], a system of confidential enquiries into maternal and perinatal morbidity and mortality [3, 4], a programme of maternity and neonatal safety investigations [5], a maternity services dashboard that provides monthly updates on the performance of maternity service providers [6], a national audit of maternity and perinatal services that regularly publishes a suite of performance indicators supporting quality improvement initiatives [7], an annual survey of women's birth experience [8], and a diverse programme of initiatives organised by professional bodies and patient charities [9, 10], among others. Additionally, there has been a series of ad‐hoc maternity investigations into maternity units. Among the recommendations from these investigations, there have been calls for meaningful and interpretable signals of quality to recognise when maternity units require intervention.

Alongside this wide palette of national programmes, there is an ongoing inspection programme carried out by the Care Quality Commission (CQC), the national independent regulator, or inspectorate, of health and social care in England [11]. The CQC publishes inspection‐informed ratings using four levels (‘outstanding, ‘good’, ‘requires improvement’, and ‘inadequate’) for five domains of quality of care (‘caring’, ‘effective’, ‘responsive’, ‘safe’, ‘well‐led’), as well as an ‘overall’ rating, for the entire hospital organisation as well as for specific services, including maternity services.

The CQC inspections involve interviews with service users and staff, observation of the clinical environment, and reviews of care records, policies, and incidents [12, 13]. The inspection framework provides a series of questions (“prompts”) for inspectors to consider alongside relevant professional standards [12]. The inspection teams also consider other relevant information from a wide range of indicators of performance across, including national clinical audits such as the National Maternity and Perinatal Audit (NMPA), to assess performance prior to an inspection [14]. Furthermore, a system of pre‐inspection risk assessment is gradually developing over time [15]. The CQC introduced a new ‘single assessment framework’ in 2023 [16].

There is ongoing debate about the clinical implications of the CQC's inspection‐informed ratings and concerns regarding the alignment of its methods with the needs of stakeholders [17]. Additionally, a review of the CQC inspection programme in 2024 highlighted issues including the lack of clarity about how ratings are calculated, a high proportion of unrated services, and inspections being conducted by personnel who lack healthcare experience [18].

In response to these critical reviews, we compared the CQC's inspection‐informed ratings with contemporaneous clinical outcomes and obstetric process measures, developed by the NMPA, derived from national administrative hospital data linked to routinely collected clinical maternity data [7]. The aim of this study is to get a better understanding of the potential role of the CQC's inspection‐informed ratings in identifying units that are in need of improvement as well as providing information to women and their families when they are making decisions about where and how they want to give birth to their baby.

## 2 Methods

### 2.1 Study Design

We used Hospital Episode Statistics (HES), administrative hospital data [19], linked to the national routinely collected Maternity Services Dataset version 1.5 (MSDS) [20]. The HES dataset provides records of all secondary care episodes provided by the English National Health Service (NHS). A HES inpatient care record contains demographic details including age and ethnic group, admission and discharge dates, neighbourhood‐based socioeconomic deprivation, and clinical information relating to the birth episode. Diagnoses are coded according to the International Classification of Diseases 10th revision (ICD‐10) [21] and procedures are coded according to the Office for Population Censuses and Surveys Classification of Interventions and Procedures 4th revision (OPCS‐4) [22]. Additional clinical information on obstetric conditions and outcomes of labour and birth is located in the ‘maternity tail’ [19]. Babies' care is recorded in the neonatal HES record. An MSDS record contains routinely collected clinical data from across the maternity pathway, also including risk factors such as maternal smoking at booking and body mass index (BMI) [23].

### 2.2 Participants

We identified women with a singleton pregnancy who gave birth at 37 or more completed weeks of gestation between 1st April 2018 and 31st March 2019. We refer to the study participants as ‘women’ but we acknowledge that some may have a different gender identity. These inclusion criteria were selected to identify a relatively homogenous population among whom differences due to maternity unit ratings may be apparent rather than differences due to either underlying biological mechanisms of complications or due to the configuration of services to support preterm/multiple births. Women were excluded if they had given birth at a freestanding midwifery unit, which are those on a geographically separate site from an obstetric unit. The numbers in these units were very low and several of these units were unrated during the study period. Women were also excluded if they were recorded to have given birth in a hospital without an established maternity service or if their records did not include a hospital identifier.

For the analysis of extended perinatal mortality, we included all babies for whom we had a neonatal HES record, and for neonatal morbidity, we included babies who were born alive and who had a neonatal HES record. For the analysis of Apgar score below 7 at 5 min of life, we included babies who were born alive and who had an MSDS record. The rate of obstetric anal sphincter injury was only determined for women who had a vaginal birth, and the rate of intrapartum caesarean birth was only for women who were in labour.

### 2.3 Outcomes

Maternal outcomes were the English Maternal Morbidity Outcome Indicator (EMMOI), a validated composite outcome for severe maternal morbidity derived from diagnostic and procedural codes in maternal HES records [24] and obstetric anal sphincter injury, defined by an ICD‐10 code for a third or fourth‐degree perineal tear or OPCS code for repair of the anal sphincter or rectal mucosa derived from HES records [25].

Neonatal outcomes were the English Neonatal Adverse Outcome Indicator (ENAOI), a validated composite outcome derived from neonatal HES records [26], extended perinatal mortality, combining stillbirth and neonatal death within 28 days after birth, and an Apgar score below 7 at 5 min of life.

Obstetric process measures were non‐spontaneous birth (i.e., either a caesarean birth before the onset of labour or an induction of labour), and intrapartum caesarean birth (i.e., caesarean birth after the onset of labour). The rationale for these measures was that both under‐or over‐intervention may represent suboptimal care that may be captured by inspections of maternity units. Definitions of the outcomes can be found in Table S1.

### 2.4 CQC Inspection‐Informed Ratings

We compared the NMPA's maternal and neonatal outcomes and the obstetric process measures according to maternity units' contemporaneous ‘overall’ CQC rating [12, 13]. The overall rating is the rating that maternity units display and that is most accessible to service users and health professionals. Few units were rated ‘inadequate’ during the study period. Therefore, the units that had the rating ‘requires improvement’ and ‘inadequate’ were combined into one group, producing a three‐level comparison: ‘outstanding’, ‘good’, and ‘requires improvement/inadequate’. Ratings were manually extracted from the CQC reports compiled within each individual maternity unit's chronological list of assessments on the CQC website for the study period. We also considered the domain‐level rating for “safe”, which includes protection from avoidable harm.

### 2.5 Women's Characteristics

Maternal demographic characteristics that were included in the risk adjustment models (see below) were maternal age (< 19, 20–24, 25–29, 30–34, 35–39, ≥ 40 years), maternal ethnic background (Black, Mixed, ‘Other’ ethnic background including Chinese, South Asian, White), and national quintiles of a neighbourhood measure of socioeconomic deprivation according to Index of Multiple Deprivation (IMD) [27] as well as birth history (nulliparous, parous without previous caesarean, parous with previous caesarean), smoking status at booking, and maternal BMI at booking according to standard World Health Organisation (WHO) categories (< 18.5, 18.5–24, 25–29, 30–34, 35–39, ≥ 40 kg/m^2^). Pregnancy risk factors were defined by ICD‐10 codes in HES and included pre‐existing diabetes, pre‐existing hypertension, gestational diabetes, pregnancy‐induced hypertension, pre‐eclampsia, and eclampsia. All definitions can be found in Table S1.

### 2.6 Characteristics of the Maternity Units

Unit characteristics were the volume of births (< 500, 500–1999, 2000–3999, ≥ 4000 births per year) and neonatal‐unit level (special‐care baby unit, local neonatal unit, neonatal intensive care unit, and neonatal surgical unit) available in the hospital organisation.

### 2.7 Statistical Analysis

Multilevel Poisson regression with maternity units included as a random effect was used to test the variation in the risk of the outcomes and the rates of process measures according to the inspection‐informed CQC ratings [28], with adjustment for characteristics of the women and the maternity units presented above. The generalised Wald test was used to test differences.

Empirical Bayes estimates were used to produce caterpillar plots of the adjusted clinical outcomes and obstetric process measures for each unit with their 95% credibility intervals [29]. Empirical Bayes estimates are considered more robust predictions of future performance than fixed‐effects estimates because the unit‐specific empirical Bayes estimates are ‘shrunk’ towards the overall mean, especially for smaller units. The caterpillar plots were used to demonstrate the variation in units' clinical outcomes and obstetric process measures within and between three inspection‐informed CQC rating groups.

We conducted three sensitivity analyses. First, we compared all study outcomes according to the CQC rating for the “safe” domain instead of the “overall” rating. Second, whilst we used the prospective maternity ratings in the main analysis (i.e., the ratings that would have been available during the study period), we also compared the study outcomes according to the ratings published closest in time to the study period even if these were retrospective (i.e., ratings available after the study period). Third, for the main analyses we included missing data on case mix variables as an additional category but we investigated the robustness of the findings to this approach, using multiple imputations with chained equations to create 10 imputed datasets [30].

All statistical analyses were conducted using Stata version 16 (StataCorp LLC, College Station, Texas).

### 2.8 Patient Involvement

There was no direct involvement of patients or the public in this research. The NMPA work is informed by the Women and Families Involvement Group (WFIG) of representatives from charity groups and lay members. The research was also informed by the perspectives of maternity service users that were systematically collected and presented across a range of maternity investigations conducted in the UK over the last 10 years and in response to the recommendations based on the experiences of service users to better understand maternity service provider signals that women may be receiving substandard care.

## 3 Results

**FIGURE 1:**
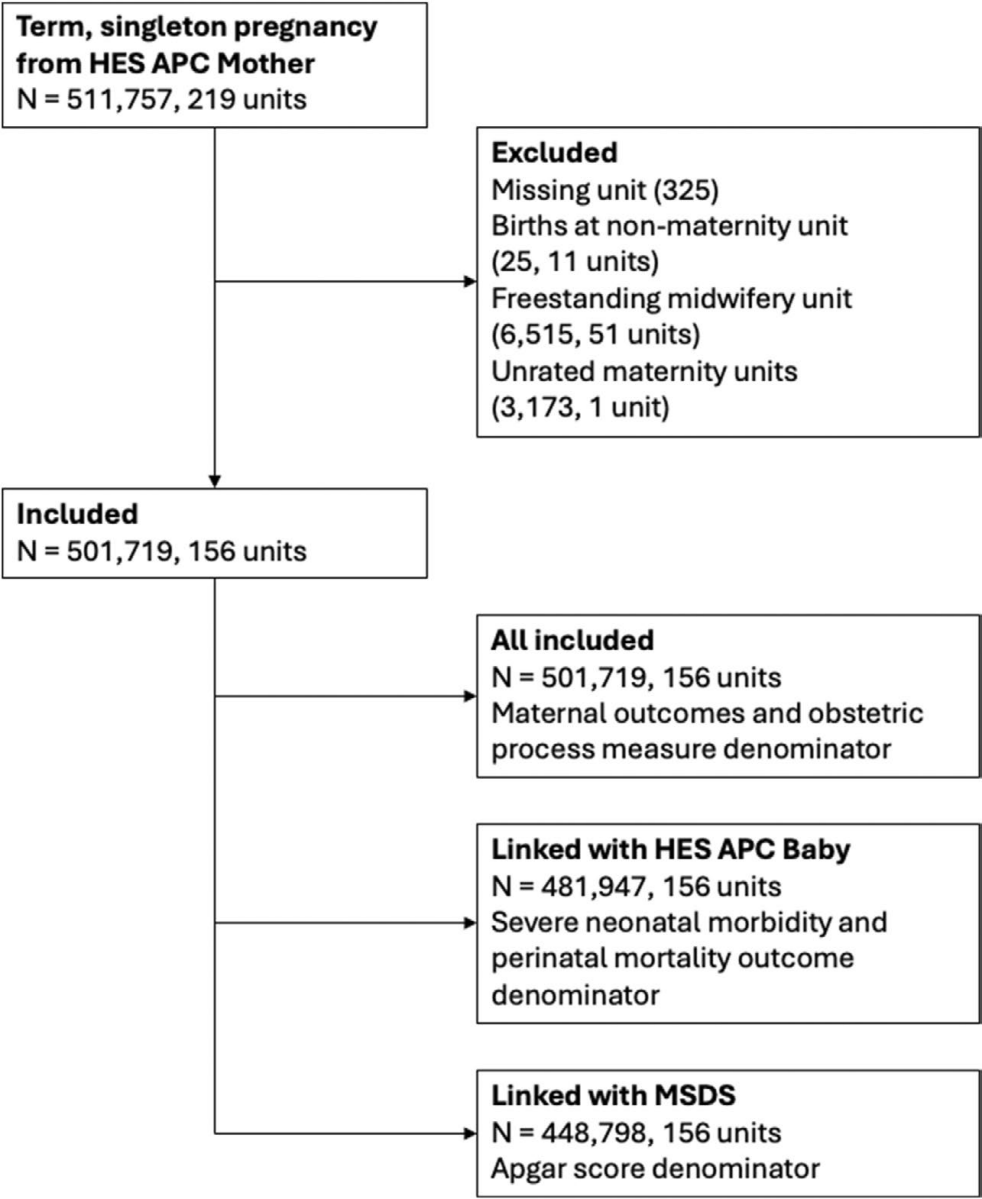
Flow Diagram of inclusion and linkages. Flow diagram of inclusion and exclusion criteria and linkages between maternal and neonatal Hospital Episode Statistics (HES) records and Maternity Services Dataset (MSDS) linkage.

We included 501 719 women with a singleton term birth in 156 maternity units (Figure 1). Of these, 39 330 (8.0%) gave births in 11 units rated as ‘outstanding’, 357 114 (71.2%) in 110 units rated as ‘good’, and 104 675 (20.9%) in 35 units rated ‘requires improvement or inadequate’. Of the 156 maternity units, 104 (66.7%) were inspected during the study period (1st April 2018 and 31st March 2019) or in the preceding 12 months.

Women who gave birth at a maternity unit rated ‘outstanding’ were typically older and more often nulliparous and from a South‐Asian, Black or ‘Other’ ethnic background, compared to women who gave birth at a maternity unit rated ‘good’ (Table 1). Women who gave birth at a maternity unit rated ‘requires improvement/inadequate’ were typically younger, more often smokers, from the most deprived neighbourhood IMD quintile, and from a White ethnic background, compared to women who gave birth at a maternity unit rated ‘good’.

**TABLE 1:** Baseline characteristics according to maternity service rating.

	Maternity service rating						Total	
	Outstanding		Good		RI/inadequate			
	*n* = 39 930		*n* = 357 114		*n* = 104 675		*n* = 501 719	
	11 units		110 units		35 units		156 units	
**Individual‐level characteristics**								
Maternal age, years								
12–19	935	2.3%	9999	2.8%	3505	3.4%	14 439	2.9%
20–24	4882	12.2%	48 952	13.7%	17 033	16.3%	70 867	14.1%
25–29	10 300	25.8%	98 387	27.6%	31 457	30.1%	140 144	27.9%
30–34	13 705	34.3%	117 931	33.0%	32 221	30.8%	163 857	32.7%
35–39	8256	20.7%	67 362	18.9%	16 897	16.1%	92 515	18.4%
≥ 40	1852	4.6%	14 483	4.1%	3562	3.4%	19 897	4.0%
Maternal ethnic group								
White	24 531	72.1%	244 149	76.4%	75 262	81.0%	343 942	77.0%
South Asian	4251	12.5%	36 986	11.6%	10 741	11.6%	51 978	11.6%
Black	1944	5.7%	16 592	5.2%	2355	2.5%	20 891	4.7%
Mixed	811	2.4%	6521	2.0%	1568	1.7%	8900	2.0%
‘Other’	2466	7.3%	15 424	4.8%	3022	3.3%	20 912	4.7%
Missing	5927	(14.8)	37 442	(10.5)	11 727	(11.2)	55 096	(11.0)
IMD, quintile								
1 (least deprived)	6403	16.0%	54 683	15.3%	13 485	12.9%	74 571	14.9%
2	6348	15.9%	62 093	17.4%	16 074	15.4%	84 515	16.9%
3	7765	19.5%	68 633	19.2%	19 794	18.9%	96 192	19.2%
4	8134	20.4%	78 204	21.9%	24 752	23.7%	111 090	22.1%
5 (most deprived)	10 895	27.3%	90 687	25.4%	30 382	29.0%	131 964	26.3%
Missing	385	(1.0)	2814	(0.8)	188	(0.2)	3387	(0.7)
Birth history								
Nulliparous	17 139	44.0%	149 194	42.7%	40 492	40.1%	206 825	42.3%
Parous, no previous CB	15 518	39.8%	146 336	41.9%	44 750	44.3%	206 604	42.2%
Parous, previous CB	6290	16.2%	53 904	15.4%	15 745	15.6%	75 939	15.5%
Missing	983	(2.5)	7680	(2.2)	3688	(3.5)	12 351	(2.5)
Body Mass Index, kg/m^2^								
< 18.5	524	2.2%	7799	2.8%	2306	2.9%	10 629	2.8%
18.5–24	10 916	45.4%	130 577	46.9%	35 470	44.3%	176 963	46.2%
25–29	6972	29.0%	79 121	28.4%	22 889	28.6%	108 982	28.5%
30–34	3379	14.1%	37 446	13.4%	11 597	14.5%	52 422	13.7%
35–39	1468	6.1%	15 497	5.6%	5000	6.3%	21 965	5.7%
≥ 40	763	3.2%	8275	3.0%	2793	3.5%	11 831	3.1%
Missing	15 908	(39.8)	78 399	(22.0)	24 620	(23.5)	118 927	(23.7)
Smoking status	3804	11.8%	37 702	12.5%	13 513	14.9%	55 019	12.9%
Missing	7669	(19.2)	54 353	(15.2)	13 724	(13.1)	75 746	(15.1)
**Unit‐level characteristics**								
Unit type								
OU	3	27.3%	29	26.4%	13	37.1%	45	28.9%
OU + AMU	8	72.7%	81	73.6%	22	62.9%	111	71.1%
Unit size								
500–1999	1	9.1%	16	14.6%	6	17.1%	23	14.7%
2000–3999	5	45.5%	47	42.7%	16	45.7%	68	43.6%
≥ 4000	5	45.5%	47	42.7%	13	37.1%	65	41.7%
NNU type								
SCBU	3	27.3%	26	23.6%	11	31.4%	40	25.6%
LNU	4	36.4%	52	47.3%	16	45.7%	72	46.2%
NICU	1	9.1%	16	14.6%	6	17.1%	23	14.7%
NICU/surgical unit	3	27.3%	16	14.6%	2	5.7%	21	13.5%

*Note:* Proportions may not sum to 100% due to rounding. Denominators for proportions exclude observations with missing data. Abbreviations: AMU, alongside midwifery unit (co‐located with an obstetric unit); CB, caesarean birth; IMD, Index of Multiple Deprivation; LNU, local neonatal unit; NICU neonatal intensive care unit; OU obstetric unit; RI requires improvement; SCBU special care baby unit.

### 3.1 Maternal Outcomes

Table 2 shows that the risk of severe maternal morbidity (EMMOI) did not vary significantly according to the CQC rating. Of the 39 930 women who gave birth in maternity units rated as ‘outstanding’, 462 (1.2%) had severe morbidity, compared to 4466 of the 357 114 (1.3%) women who gave birth in units rated ‘good’, and 1041 of the 104 675 (1.0%) women in units rated ‘requires improvement/inadequate’ (adjusted *p* = 0.59). Neither was there significant variation in the risk of obstetric anal sphincter injury. Of the 27 567 women who gave birth vaginally in units rated as ‘outstanding’, 905 (3.3%) had an obstetric anal sphincter injury, compared to 8031 of the 255 034 (3.1%) in units rated ‘good’ and 2336 of the 75 412 (3.1%) in units rated ‘requires improvement/inadequate’ (adjusted *p* = 0.75). The models from which the adjusted *p*‐values were obtained are shown in Table S2, with relative risks and 95% confidence intervals, comparing women who gave birth in units rated as ‘outstanding’ or as ‘requires improvement/inadequate’ with women who gave birth in units rated as ‘good’.

**TABLE 2:** Maternal and neonatal outcomes according to overall maternity service rating.

	Maternity service rating						Total	
	Outstanding		Good		RI/inadequate			
	*n* = 39 930		*n* = 357 114		*n* = 104 675		*n* = 501 719	
		% (95% CI)		% (95% CI)		% (95% CI)		% (95% CI)
Maternal outcome								
EMMOI	462/39930	1.2 (0.87–1.5)	4466/357114	1.3 (1.1–1.4)	1041/104675	1.0 (0.87–1.1)	5969/501719	1.2 (1.1–1.3)
OASI	905/27567	3.3 (2.7–4.0)	8031/255034	3.1 (3.0–3.3)	2336/75412	3.1 (2.8–3.4)	11272/358013	3.1 (3.0–3.3)
Neonatal outcome								
EPNM	52/38144	0.14 (0.10–0.18)	484/340986	0.14 (0.12–0.16)	162/102817	0.16 (0.13–0.19)	698/481947	0.14 (0.13–0.16)
ENAOI	1634/38112	4.3 (3.3–5.6)	13 782/340667	4.0 (3.6–4.5)	3462/102707	3.4 (2.9–3.9)	18 878/481486	3.9 (3.6–4.3)
Apgar < 7 at 5 min	442/36494	1.2 (0.81–1.8)	3403/331033	1.0 (0.95–1.1)	1026/95174	1.1 (0.93–1.3)	4871/462701	1.1 (0.98–1.1)
Process measure								
Non‐spontaneous birth	16 866/35099	48.1 (42.2–53.9)	149 171/311695	47.9 (46.4–49.4)	42 109/87882	47.9 (45.1–50.8)	208 146/434676	47.9 (46.6–49.2)
Intrapartum caesarean	5673/33726	16.8 (14.6–19.3)	50 885/307222	16.6 (15.8–17.3)	14 218/90048	15.8 (14.9–16.7)	70 776/430996	16.4 (15.8–17.0)

*Note:* The denominators for obstetric anal sphincter injury, ENAOI, EPNM, and Apgar < 7 at 5 min were: vaginal birth, live birth, babies with linkage to HES APC neonatal record, and linkage to the MSDS record, respectively.

Abbreviations: EMMOI, English Maternal Morbidity Outcome Indicator; ENAOI, English Neonatal Adverse Outcome Indicator; EPNM, Extended perinatal mortality; OASI, obstetric anal sphincter injury; RI, requires improvement.

### 3.2 Neonatal Outcomes

We found no significant variation in the combined risk of stillbirth and neonatal mortality (i.e., extended perinatal mortality), severe neonatal morbidity (ENAOI), or an Apgar score below 7 at 5 min according to the CQC rating (Table 2). Of the 38 144 included babies born in maternity units rated as ‘outstanding’, 52 (0.14%) were stillborn or died within 28 days, compared to 484 of the 340 986 (0.14%) born in units rated ‘good’ and 162 of the 102 817 (0.16%) born in units rated ‘requires improvement/inadequate’ (adjusted *p* = 0.32). Of the 38 112 live births in units rated as ‘outstanding’, 1634 (4.3%) babies had severe neonatal morbidity, compared to 13 782 of the 340 667 (4.0%) babies in units rated ‘good’ and 3462 of the 102 707 (3.4%) babies in units rated ‘requires improvement/inadequate’ (adjusted *p* = 0.48). Of the 36 494 live births with an Apgar score at 5 min in units rated as ‘outstanding’, 442 (1.2%) had a score below 7, compared to 3403 of the 331 003 (1.0%) born in units rated ‘good’ and 1026 of the 95 174 (1.1%) born in units rated ‘requires improvement/inadequate’ (adjusted *p* = 0.53). Model results are shown in Table S2.

### 3.3 Obstetric Process Measures

We found no variation in non‐spontaneous birth, which occurred in 16 866 of the 35 099 (48.1%) births in units rated as ‘outstanding’, 149 171 of the 311 695 (47.9%) births in units rated ‘good’, and 42 109 of the 87 882 (47.9%) births in units rated ‘requires improvement/inadequate’ (adjusted *p* = 0.87). Nor did we find variation in the rate of intrapartum caesarean birth, which occurred in 5673 of the 33 726 (16.8%) births in units rated as ‘outstanding’, 50 885 of the 307 222 (16.6%) births in units rated ‘good’, and 14 218 of the 90 048 (15.8%) births in the units rated ‘requires improvement/inadequate’ (adjusted *p* = 0.87). Model results are shown in Table S2.

### 3.4 Variation Between Maternity Units

**FIGURE 2:**
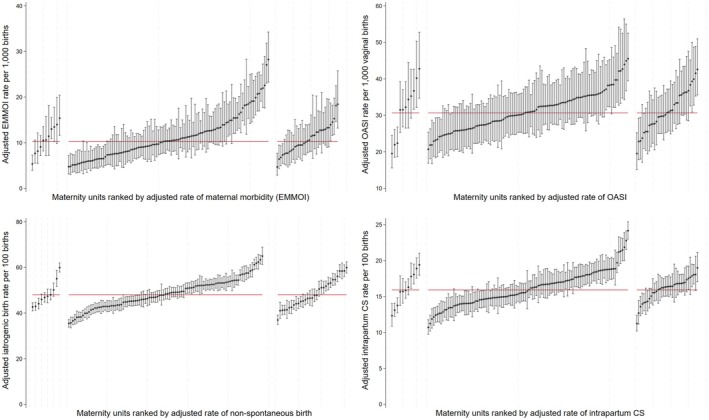
Ranked empirical Bayes estimates for maternal outcomes. Within each plot, units rated ‘outstanding’ are shown on the left, ‘good’ are shown in the middle, ‘requires improvement/inadequate’ are shown on the right. The red horizontal line represents the national average.

**FIGURE 3:**
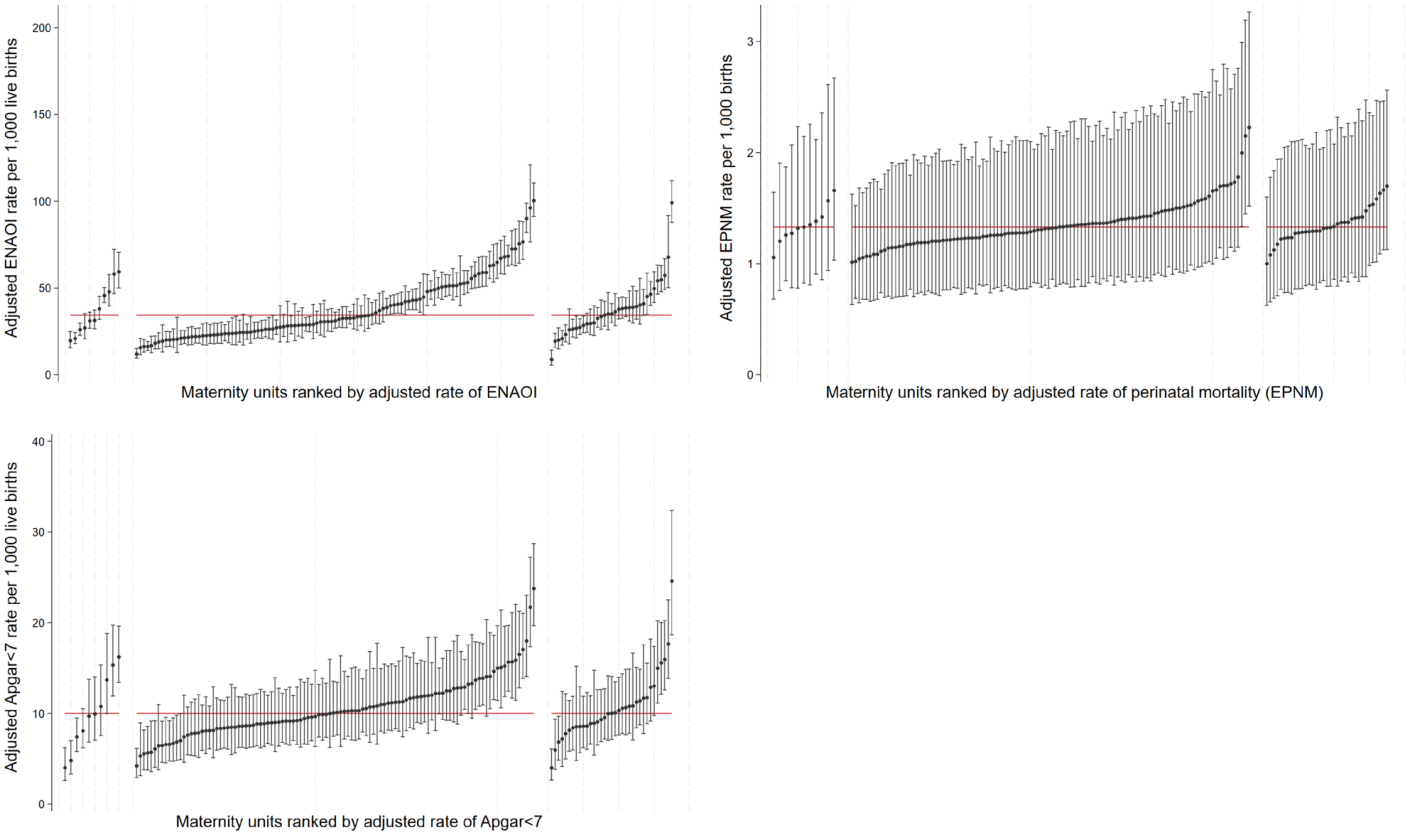
Ranked empirical Bayes estimates for neonatal outcomes. Within each plot, units rated ‘outstanding’ are shown on the left, ‘good’ are shown in the middle, ‘requires improvement/inadequate’ are shown on the right. The red horizontal line represents the national average.

The caterpillar plots of the adjusted empirical Bayes estimates for maternal outcomes (Figure 2) and neonatal outcomes (Figure 3) demonstrate the considerable variation in clinical outcomes and obstetric process measures among the maternity units within each of the rating categories. However, these plots also demonstrate that the differences across the rating categories are small, both in terms of the overall mean as well as the variation between the units.

### 3.5 Sensitivity Analyses

Repeat analyses comparing the clinical outcomes and the obstetric process measures according to the CQC's ‘safe’ domain rating produced similar results (see for detailed results Table S3). Additionally, when we used only the ratings closest in time to the study period, even if these were retrospective, we identified 126 units that were rated within 12 months of the study period and only 6 had different inspection‐informed ratings compared to the main analysis. The rate of intrapartum caesarean birth differed prior to adjustment but not following adjustment (Table S4). No other indicators differed. Lastly, the use of multiple imputations to handle missing data did not change the results.

## 4 Discussion

### 4.1 Main Findings

This study did not find systematic differences in clinical outcomes and obstetric practice measures among English NHS maternity units according to the units' ‘overall’ inspection‐informed rating published by the CQC, the national health and social care regulator in England. Neither were there systematic differences among the maternity units according to their rating of being ‘safe’.

### 4.2 Strengths and Limitations

The first strength of this research is its national coverage of NHS maternity services, providing more than 99% of maternity care in England [31]. Second, the national maternity dataset provides rich clinical data [23], in addition to the robust information on maternity services available in the administrative hospital data [32]. Third, the use of routinely collected data bypasses the reliance on clinician reporting and, as such, reduces the recognised risk of under‐reporting of inappropriate care processes or adverse events in clinical research registries [33, 34].

The first limitation is that we compared clinical outcomes and obstetric practice measures of births that took place between April 2018 and March 2019 against inspection‐based ratings that were published at that time. As a result, we do not fully capture the subsequent changes in the CQC's inspection system, especially related to the role of pre‐inspection risk assessments [15]. However, the fundamental principles of CQC's inspection approach have not changed since 2018. We did not use a more recent inclusion period to avoid the deterioration in the quality and completeness of essential data items in the years immediately after the impact of the introduction of a new version of the national maternity services dataset in April 2019 [35] and the impact of the COVID‐19 pandemic on clinical outcomes and obstetric practice [36].

A second potential limitation is that some of the observed variations between the maternity units, especially the composite measures, may be explained by the quality of clinical coding at individual hospitals. However, we have previously demonstrated a high level of consistency in relevant obstetric procedure codes across hospital organisations in the English NHS [32]. These administrative data depend on both clinical diagnosis and coding and so under‐diagnosis may also contribute to variation [34], for example, for perineal injury, although there was no evidence of potential under‐diagnosis according to unit rating. Further limitations are missing data and the lack of linkage of some records of maternity episodes with the other administrative data. It is unlikely that it affected our study outcomes because a sensitivity analysis using multiple imputation for missing data did not change the results appreciably.

Lastly, severe haemorrhage is represented in the EMMOI only through management criteria or other end‐organ complications. We could not include severe haemorrhage as a separate outcome because in none of the sources of data was it possible to distinguish severe from non‐severe haemorrhage. The measures represent potentially modifiable complications related to physical health. Whilst these are not the only dimensions of care that CQC inspections consider, preventable maternal and neonatal morbidity and mortality are the focus of the majority of quality assurance processes and have been at the centre of every maternity investigation.

### 4.3 Interpretation

A previous study of inspection‐informed ratings of maternity units of the English NHS published by the CQC found limited evidence that clinical practice prior to inspection, derived only from administrative hospital data for birth between 2013 and 2016, differed according to whether the subsequent ratings were positive or negative [37]. As a result, the study called into question the validity of the inspection‐informed ratings, the reliability of clinical outcomes and obstetric process measures derived from administrative hospital data, or both. Our study builds on that previous study by linking to national routinely collected clinical maternity data, by including more recent births, and by only using validated clinical outcomes and obstetric process measures that are trialled and tested by the NMPA [7]. Consequently, we argue that it is unlikely that problems with the validity of the outcome and process measures explain the lack of association between inspection‐informed ratings of maternity units published by the national healthcare regulator. Moreover, as argued above, our own work has already demonstrated the appropriateness of using administrative hospital data to evaluate maternity services, even without linkage to routinely collected clinical maternity data [32]. Given our observation that women from the most deprived neighbourhoods were more likely to give birth in a maternity unit rated ‘requires improvement/inadequate’, the inspection‐informed ratings may reflect the characteristics of the population that the units serve rather than the safety and quality of the care that they provide.

We compared the inspection‐informed ratings published by the CQC with obstetric process measures that were chosen as complementary indicators of a maternity unit's ‘practice style’, considering that the rate of ‘non‐spontaneous birth’ reflects the extent to which hospitals provide ‘proactive care’ for women with term pregnancies and that the rate of ‘emergency caesarean birth’ for women in labour reflects how services respond acutely to provide ‘immediate preventive action’ or ‘rescue’. Whilst there is no target rate for these interventions, practice style is associated with clinical outcomes [38] and variation nevertheless may reflect clinical under‐ or over‐intervention. We found considerable variation among the maternity units within the three inspection‐informed CQC rating groups but little variation between them, which suggests that these ratings do not capture major variations in the units' obstetric practice style.

All clinical outcome measures used in this study can be considered as measures of potentially avoidable harm to mother and baby or, conversely, measures of safe obstetric practice. A sensitivity analysis showed that there is also no association between these clinical outcomes and the inspection‐informed ratings of the ‘safe’ domain.

Ratings have previously been criticised because they are based on limited information and sometimes obtained from inspectors who lack relevant expertise [18] and whose ratings may disagree among themselves [39]. It has also been demonstrated that a wide range of relevant ‘intelligent monitoring’ performance indicators, including the results of the National Maternity and Perinatal Audit, selected by the CQC do not predict the inspection‐informed ratings of units in primary and secondary care outside the context of maternity [40, 41]. Further research is needed to get a better understanding of the associations between the inspection‐informed ratings and other indicators of healthcare quality, including patient experience measures [42]. There is also a need to identify the most efficient system to keep the published inspection‐informed ratings up‐to‐date.

A first policy implication of this study is that its results suggest that inspection‐informed ratings reflect neither clinical outcomes nor obstetric practice related to the care of singleton pregnancies at term in maternity units in the English NHS. As a result, the ratings published by the CQC may lead either to ‘false alarms’ (i.e., labelling a unit as ‘requires improvement/inadequate’ whilst its outcomes and process measures are not systematically different from units labelled as ‘good’ or ‘outstanding’) or conversely to ‘false reassurance’ (labelling a unit as ‘good’ or ‘outstanding’ whilst its outcomes and process measures are not systematically different from units labelled as ‘requires improvement/inadequate’) if service users and stakeholders interpret the ratings to reflect clinical outcomes or obstetric practice. These inspection‐informed ratings cannot be relied upon to identify underperforming maternity units. Conversely, data from national surveillance of perinatal mortality in England may be able to identify under‐performance [43]. We would argue clinical outcomes ought to be central to this evaluation.

Second, reviews of the CQC inspection programme, including the one carried out in 2024 [18], should focus on the validity and reliability of the CQC's ratings. This implies that the ongoing development of the regulatory inspection programme should be embedded in an extensive programme of research that aims to improve the validity and reliability of the published ratings [16]. For example, a greater role could be given to validated outcome and process measures published by other national initiatives [6, 7, 44, 45, 46]. This may also apply to inspection programmes across the broader range of services that NHS hospitals provide.

Third, one major criticism of the inspection‐informed ratings system is that in some cases the most up‐to‐date ratings were ‘historic’ given that they were based on inspections that took place several years in the past [18]. However, our sensitivity analysis only including units that were rated within 12 months did not change our findings, which suggests that simply improving the timeliness of the published ratings will not have a major impact on their validity.

Fourth and most fundamental, the assessment of a hospital organisation at one point in time by a small inspection team may not provide a meaningful measure of the quality of the care it provides, particularly for rare clinical outcomes. Assessments may be strengthened if the inspection teams were to focus on gathering experiential and cultural information from meeting staff and service users [14], focusing on domains such as “caring” and “well‐led” and evaluating clinical outcomes using comprehensive and objective sources of data [47]. How these different sources of information, including on outcomes and patient experience, are selected and synthesised should be informed by the perspectives of stakeholders who make use of the assessments.

## 5 Conclusions

There was no clear pattern of association between inspection‐informed ratings of maternity units published by the English national healthcare regulator and clinical outcomes and obstetric practice measures derived from routinely collected data. Concerted action is needed to improve the inspection‐informed ratings of maternity services so that quality assurance and improvement initiatives and women's choices of how and where they want to give birth are guided by clinically relevant evidence.

## Author Contributions

The study was conceived and designed by A.K., J.v.d.M., S.O., I.G.‐U., and I.H., with feedback from all the authors. I.H. performed the statistical analysis with validation by I.G.‐U. I.H. wrote the first draft of the manuscript. All authors critically reviewed the manuscript and provided final approval of the submitted manuscript.

## Disclosure

All individuals, apart from I.H. and J.v.d.M., are or have been partially or wholly funded by the Healthcare Quality Improvement Partnership for their contribution to the submitted work. All authors declare no financial relationships with any organisation that might have an interest in the submitted work in the previous 3 years. A.K. is the Vice President of the Royal College of Obstetricians and Gynaecologists. A.M. was a research midwife for the National Perinatal Epidemiology Unit at the University of Oxford during the conduct of this study. The other authors report no other relationships or activities that could appear to have influenced the submitted work.

## Ethics Statement

Approval for the use of pseudonymised personal health data without consent was granted by the NHS Health Research Authority (16/CAG/0058). This national audit investigated variation in clinical care and was exempt from ethical review by the NHS Health Research Authority.

## Conflicts of Interest

A.K. is Vice President of the Royal College of Obstetricians and Gynaecologists. A.M. was employed by the National Perinatal Epidemiology Unit during the conduct of this study. All other authors declare no conflicts of interest.

## Supporting information


**Table S1.** Definitions of case‐mix variables and outcomes.
**Table S2.** Association between Maternity Service “Overall” Rating and Outcomes.
**Table S3.** Association between Maternity Service “Safe” Rating and Outcomes [sensitivity analysis].
**Table S4.** Association between Maternity Service Restricted Ratings and Outcomes [sensitivity analysis].

## Data Availability

The data that support the findings of this study are available from the Healthcare Quality Improvement Partnership. Restrictions apply to the availability of these data. Information on how to access the data is available from https://www.hqip.org.uk/national‐programmes/accessing‐ncapop‐data/.
