# Supplementary material for: A Comparison of Regulatory Maternity Unit Ratings With Clinical Outcomes and Practice Measures: An Observational Study Using Routinely Collected Data
Source: BJOG. 2025 May 5;132(9):1285–96. doi: 10.1111/1471-0528.18188 (PMC12232518; doi:10.1111/1471-0528.18188)
Supplement: Supplementary file 1 — Table S1. Definitions of case‐mix variables and outcomes. Table S2. Association between Maternity Service “Overall” Rating and Outcomes. Table S3. Association between Maternity Service “Safe” Rating and Outcomes [sensitivity analysis]. Table S4. Association between Maternity Service Restricted Ratings and Outcomes [sensitivity analysis]. [file BJO-132-1285-s001.docx]

SUPPLEMENTARY INFORMATION

**A comparison of maternity unit ratings informed by regulatory inspections with clinical outcomes and obstetric practice measures derived from routinely collected data in England**

Henderson I et al.

CONTENT:

1. Supplementary Table S1: Definitions of Case-mix Variables and Outcomes
2. Supplementary Table S2: Association between Maternity Service “Overall” Rating and Outcomes
3. Supplementary Table S3: Association between Maternity Service “Safe” Rating and Outcomes [sensitivity analysis]
4. Supplementary Table S4: Association between Maternity Service Restricted Ratings and Outcomes [sensitivity analysis]

CORRESPONDENCE:

Ipek Gurol-Urganci

London School of Hygiene and Tropical Medicine, London, UK

Email: ipek.gurol@lshtm.ac.uk

| **Supplementary Table 1** Definitions of a maternity episode, Case-mix Variables and Outcomes   \| **Definition of a maternity episode in Hospital Episode Statistics (HES) Admitted Patient Care (APC)** \| \| \| \| \| \| --- \| --- \| --- \| --- \| --- \| \| A maternity episode was defined as any record that contained valid information about mode of birth in either the procedure fields (OPCS-4 codes: R17.1 to R25.9) or in the HES maternity tail. Births before 37 completed weeks of gestation and multiple births (ICD-10 codes: Z37.2–Z37.7) or strong evidence of a multiple birth in the HES maternity tail (e.g., presence two or more distinct recorded birthweights) were excluded. \| \| \| \| \| \|  \| \| \| \| \| \| **Definition of case-mix variables and outcomes** \| \| \| \| \| \| **Measure** \| \| **Numerator/definition** \| \| **Denominator** \| \| **Maternal risk adjustment** \| \| \| \| \| \| Maternal age \| <19, 20-24, 25-29, 30-34, 35-39, ≥40 years, based on maternal age as identified in Hospital Episode Statistics (HES) Admitted Patient Care (APC) record. \| \| NA \| \| \| Index of Multiple Deprivation \| Quintile of IMD according to ‘lower super output area’, identified in HES. \| \| NA \| \| \| Parity and previous caesarean birth \| Identified in the HES maternity tail. If missing in the maternity tail, the ‘look back’ approach using previous HES records was used. \| \| NA \| \| \| Maternal ethnic group \| Asian (Bangladeshi, Indian, Pakistani, other Asian); Black (African, Caribbean, Black other); ‘Other’ (Chinese, other ethnic groups); Mixed (multiple ethnic identities); White (British, Irish, White other), identified in HES. \| \| NA \| \| \| Smoking status at booking \| Smoker or non-smoker at booking, identified in MSDS. \| \| NA \| \| \| Maternal body mass index \| BMI at booking <18.5, 18.5-24, 25-29, 30-34, 35-39, ≥40 kg/m^2^, identified in MSDS. \| \| NA \| \| \| Pre-existing hypertension \| E10, E11, E13, E14, O24.0, O24.1, O24.2, O24.3 \| \| NA \| \| \| Gestational hypertension \| O13 \| \| NA \| \| \| Pre-eclampsia \| O14 \| \| NA \| \| \| Pre-existing diabetes mellitus \| O24.0, O24.1, O24.2, O24.3 \| \| NA \| \| \| Gestational diabetes mellitus \| O24.4 \| \| NA \| \| \| Obstetric cholestasis \| O26.6 \| \| NA \| \| \| **Maternal indicators** \| \| \| \| \| \| Obstetric anal sphincter injury \| \| Third degree and fourth degree perineal tear defined by ICD codes O70.2 and O70.3, respectively, or repair of anal sphincter procedural codes R32.2 and R32.5; including button-hole injury to anal mucosa by ICD code O70.04. \| \| Vaginal births \| \| English maternal morbidity outcome indicator (EMMOI) \| \| **Diagnoses:** acute abdomen, acute renal failure, cardiac arrest/failure/infarction, cerebral oedema/coma, disseminated intravascular coagulopathy, cerebrovascular accident, major complications of anaesthesia, amniotic fluid embolism/venous thromboembolism, shock, sickle crisis, status asthmaticus, status epilepticus, uterine rupture, eclampsia, sepsis, cerebral venous thrombosis  **Procedures**: ventilation, curettage under general anaesthetic, renal dialysis, evacuation of hematoma, hysterectomy, surgical management of haemorrhage, re-closure of disrupted caesarean wound, repair of bladder or cystostomy and repair of intestine.  Coding of diagnoses and procedures can be found at Nair M, Kurinczuk JJ, Knight M. Establishing a National Maternal Morbidity Outcome Indicator in England: A Population-Based Study Using Routine Hospital Data. PloS one. 2016;11(4):e0153370 \| \| All births \| \| Spontaneous onset of labour \| \| **Spontaneous onset of labour** (HES delivery onset *delonset* value 1) \| \| NA \| \| **Or emergency caesarean ‘prior to labour’** (R18/R251 + *delonset* value 2) **and indication of labour** (O63.0, O63.1, O64, O65, O66, O62.1 [prolonged first stage of labour, prolonged second stage of labour, obstructed labour, arrested active phase of labour]) \| \| \| If missing then: \| \| \| **Or indication of labour** (as above) **without augmentation/induction** (R14, R15 [surgical induction/augmentation, medical induction/augmentation])) \| \| \| **Or unassisted vaginal birth** (ICD O80, R23, R24, R201) **without augmentation/induction** (as above) \| \| \| **Or assisted vaginal birth** (R202, R21, R22) **without augmentation/induction** (as above)  **and with admission on the day of or the day prior to birth and gestational age 38+0 to 41+6** **and without indication for induction of labour** (O10, O13, O14, O15, O24.0, O24.1, O24.4, O36.5, O36.6, O40, O41.0, O26.6 [hypertensive diseases, blood glucose disorders, suspected abnormalities of fetal growth rate, oligohydramnios, polyhydramnios, biliary disease], or maternal age ≥40 years) \| \| \| **Or assisted vaginal birth** (as above) **admitted on the day of birth** \| \| \| Non-spontaneous birth \| \| **Induction of labour** HES *delonset* values 3-5 (surgical induction, medical induction, or combination of surgical and medical induction) \| \| All births \| \| **or ‘birth prior to labour’** HES *delonset* value 2 (caesarean birth) **without indication of labour** (as above) **and without induction/augmentation** (R14, R15) \| \| \| If missing then: \| \| \| **vaginal birth** (R202, R21, R22, R23, R24) **after augmentation/induction** (R14, R15) **admitted >2 days prior to birth** \| \| \| **Or vaginal birth** **after augmentation/induction** (as above) **admitted >1 day prior to birth with either clinical indication for induction of labour** (O10, O13, O14, O15, O24.0, O24.1, O24.4, O36.5, O36.6, O40, O41.0, O26.6, or maternal age ≥40 years) **or gestational age <39 completed weeks** \| \| \| **Or emergency caesarean** (R18, R251) **after augmentation/induction** (as above) **and indication of labour** (as above) **admitted >2 days prior to birth** \| \| \| Intrapartum caesarean \| \| **Or emergency caesarean after augmentation/induction** (as above) **and indication of labour** (as above) **admitted >1 day prior to birth with clinical indication for induction of labour** (as above) \| \| Either spontaneous or non-spontaneous onset of labour \| \| **Any caesarean birth** (R17, R18, R201) **with either indication of labour** (O10, O13, O14, O15, O24.0, O24.1, O24.4, O36.5, O36.6, O40, O41.0, O26.6 [hypertensive diseases, blood glucose disorders, suspected abnormalities of fetal growth rate, oligohydramnios, polyhydramnios, biliary disease], or maternal age ≥40 years) **or after** **spontaneous onset of labour** (above) \| \| \|  \| \| **Or emergency caesarean** (R18, R201) **after non-spontaneous onset of labour and not caesarean prior to labour** (*delonset* value 2) \| \|  \| \| **Neonatal indicators** \| \| \| \| \| \| Apgar <7 at 5 minutes \| \| Recorded in MSDS. \| \| Live births with linkage to MSDS \| \| Extended perinatal mortality (EPNM) \| \| Recorded in MSDS. \| \| Live births with linkage to HES APC Baby \| \| English neonatal adverse outcome indicator (ENAOI) \| \| Stillbirth or neonatal death occurring up to 28 days of life. Recorded in MSDS. \| \| Births with linkage to HES APC Baby \| \|  \| \| **Diagnoses:** birthweight <1500 g, 28-day neonatal mortality, respiratory distress syndrome, seizure, intraventricular haemorrhage (grade 3/4), cerebral infarction, periventricular leukomalacia, birth trauma, hypoxic ischemic encephalopathy, necrotizing enterocolitis, sepsis/septicaemia, pneumonia, respiratory disease, bacterial meningitis  **Procedures**: resuscitation, mechanical ventilation/CPAP/high flow nasal oxygen, central venous or arterial catheter, pneumothorax requiring intercostal catheter, any intravenous fluids, any body cavity surgical procedure, therapeutic hypothermia.  Gestational age <32 completed weeks was omitted in this sample of women who gave birth at term.  Coding of diagnoses and procedures can be found at Knight HE, Oddie SJ, Harron KL, Aughey HK, van der Meulen JH, Gurol-Urganci I, et al. Establishing a composite neonatal adverse outcome indicator using English hospital administrative data. Arch Dis Child Fetal Neonatal Ed. 2019;104(5):F502-9 \| \|  \| \|  \| \| Stillbirth: Defined using ICD-10 codes (Z37·1) or birth status field (birstat_1=2,3,4) in the HES maternity tail for providers with over 95% data completeness. In the United Kingdom stillbirth is defined as birth without signs of life occurring at or after 24+0 completed gestational weeks, based on estimated due date calculated using universally offered ultrasound scan at 11–13 weeks’ gestation.  28-day neonatal mortality: Derived from Office for National Statistics (ONS) Mortality Data linked to HES \| \|  \| |
| --- | --- | --- | --- | --- | --- | --- | --- | --- | --- | --- | --- | --- | --- | --- | --- | --- | --- | --- | --- | --- | --- | --- | --- | --- | --- | --- | --- | --- | --- | --- | --- | --- | --- | --- | --- | --- | --- | --- | --- | --- | --- | --- | --- | --- | --- | --- | --- | --- | --- | --- | --- | --- | --- | --- | --- | --- | --- | --- | --- | --- | --- | --- | --- | --- | --- | --- | --- | --- | --- | --- | --- | --- | --- | --- | --- | --- | --- | --- | --- | --- | --- | --- | --- | --- | --- | --- | --- | --- | --- | --- | --- | --- | --- | --- | --- | --- | --- | --- | --- | --- | --- | --- | --- | --- | --- | --- | --- | --- | --- | --- | --- | --- | --- | --- | --- | --- | --- | --- | --- | --- | --- | --- | --- | --- | --- | --- | --- | --- | --- | --- | --- | --- | --- | --- | --- | --- | --- | --- | --- | --- | --- | --- | --- | --- | --- | --- | --- | --- | --- | --- | --- | --- | --- | --- | --- | --- | --- | --- | --- | --- | --- | --- | --- | --- | --- | --- | --- | --- | --- | --- | --- | --- | --- | --- | --- | --- | --- | --- | --- |

**Supplementary Table S2** Association between Maternity Service “Overall” Rating and Outcomes

|  | **Maternity Service Rating** | | |  | |
| --- | --- | --- | --- | --- | --- |
|  | **Outstanding** | **Good** | **RI/Inadequate** |  | |
|  | RR (95% CI) |  | RR (95% CI) | P-value | |
| **Maternal outcome** |  |  |  |  | |
| EMMOI (n=501 719) | n = 39 930 | n = 357 114 | n = 104 675 |  | |
| Unadjusted | 1.00 (0.74-1.36) | ref | 0.86 (0.71-1.04) | 0.31 | |
| Adjusted case-mix & unit char | 0.93 (0.69-1.24) | ref | 0.92 (0.76-1.10) | 0.59 | |
|  |  |  |  |  | |
| OASI (n=358,013) | n = 27 567 | n = 255 034 | n = 75 412 |  | |
| Unadjusted | 1.08 (0.93-1.27) | ref | 0.96 (0.87-1.05) | 0.33 | |
| Adjusted case-mix & unit char | 1.04 (0.89-1.20) | ref | 1.03 (0.94-1.14) | 0.75 | |
|  |  |  |  |  | |
| **Neonatal outcome** |  |  |  |  | |
| EPNM (n=481,947) | n = 38 144 | n = 340 978 | n = 102 825 |  | |
| Unadjusted | 0.99 (0.68-1.44) | ref | 1.08 (0.86-1.37) | 0.79 | |
| Adjusted case-mix & unit char | 0.88 (0.61-1.25) | ref | 1.15 (0.92-1.43) | 0.32 | |
|  |  |  |  |  | |
| ENAOI (n=481,486) | n = 38 112 | n = 340 667 | n = 102 707 |  | |
| Unadjusted | 1.15 (0.85-1.56) | ref | 0.88 (0.73-1.06) | 0.21 | |
| Adjusted case-mix & unit char | 1.08 (0.81-1.46) | ref | 0.91 (0.76-1.09) | 0.48 | |
|  |  |  |  |  | |
| Apgar <7 at 5 minutes (n=462,701) | n = 36 494 | n = 331 033 | n = 95 174 |  | |
| Unadjusted | 1.17 (0.90-1.51) | ref | 1.05 (0.89-1.24) | 0.46 | |
| Adjusted case-mix & unit char | 1.14 (0.88-1.47) | ref | 1.06 (0.90-1.24) | 0.53 | |
|  |  |  |  |  | |
| **Process measure** |  |  |  |  | |
| Non-spontaneous birth (n=434,676) | n = 35 099 | n = 311 695 | n = 87 882 |  | |
| Unadjusted IRR | 1.01 (0.92-1.11) | ref | 1.00 (0.94-1.06) | 0.98 | |
| Adjusted case-mix & unit char | 1.00 (0.92-1.10) | ref | 0.99 (0.93-1.04) | 0.87 | |
|  |  |  |  |  | |
| Intrapartum caesarean (n=430,996) | n = 33 726 | n = 307 222 | n = 90 048 |  | |
| Unadjusted | 1.04 (0.93-1.17) | ref | 0.97 (0.91-1.05) | 0.57 | |
| Adjusted case-mix & unit char | 1.00 (0.91-1.10) | ref | 1.02 (0.96-1.08) | 0.87 | |
|  |  |  |  |  | |
| Case-mix adjustment: maternal age and ethnic background, IMD quintile, body mass index, smoking status, pre-existing hypertension, pregnancy-induced hypertension, pre-eclampsia, gestational diabetes, diabetes, obstetric cholestasis. | | | | |  |
| Unit characteristics: unit delivery volume and neonatal until type | | | | |  |

**Supplementary Table S3** Association between Maternity Service “Safe” Domain Rating and Outcomes

|  | **Maternity Service Rating for 'Safe'** | | |  | | |  |
| --- | --- | --- | --- | --- | --- | --- | --- |
|  | **Outstanding/Good** |  | **RI/Inadequate** | | |  |  |
|  | RR (95% CI) |  | RR (95% CI) | | P-value | |  |
| **Maternal outcome** |  |  |  | |  | | |
| EMMOI (n=501 719) | n = 266 012 |  | n = 235 707 | |  | | |
| Unadjusted | ref |  | 0.91 (0.78-1.07) | | 0.25 | | |
| Adjusted case-mix & unit char | ref |  | 0.92 (0.79-1.07) | | 0.27 | | |
|  |  |  |  | |  | | |
| OASI (n=358,013) | n = 189 550 |  | n = 168 463 | |  | | |
| Unadjusted | ref |  | 0.95 (0.88-1.03) | | 0.19 | | |
| Adjusted case-mix & unit char | ref |  | 0.96 (0.88-1.04) | | 0.31 | | |
|  |  |  |  | |  | | |
| **Neonatal outcome** |  |  |  | |  | | |
| EPNM (n=481,947) | n = 255 483 |  | n = 226 464 | |  | | |
| Unadjusted | ref |  | 0.98 (0.81-1.19) | | 0.85 | | |
| Adjusted case-mix & unit char | ref |  | 1.00 (0.83-1.21) | | 0.99 | | |
|  |  |  |  | |  | | |
| ENAOI (n=481,486) | n = 255 240 |  | n = 226 246 | |  | | |
| Unadjusted | ref |  | 1.06 (0.91-1.24) | | 0.47 | | |
| Adjusted case-mix & unit char | ref |  | 1.06 (0.92-1.24) | | 0.41 | | |
|  |  |  |  | |  | | |
| Apgar <7 at 5 minutes (n=462,701) | n = 245 191 |  | n = 217 510 | |  | | |
| Unadjusted | ref |  | 0.93 (0.81-1.07) | | 0.34 | | |
| Adjusted case-mix & unit char | ref |  | 0.96 (0.84-1.10) | | 0.55 | | |
|  |  |  |  | |  | | |
| **Process measure** |  |  |  | |  | | |
| Non-spontaneous birth (n=434,676) | n = 225 438 |  | n = 209 238 | |  | | |
| Unadjusted IRR | ref |  | 0.98 (0.93-1.03) | | 0.44 | | |
| Adjusted case-mix & unit char | ref |  | 0.99 (0.94-1.03) | | 0.62 | | |
|  |  |  |  | |  | | |
| Intrapartum caesarean (n=430,996) | n = 227 189 |  | n = 203 807 | |  | | |
| Unadjusted IRR | ref |  | 1.05 (0.99-1.11) | | 0.15 | | |
| Adjusted case-mix & unit char | ref |  | 1.05 (1.00-1.10) | | 0.06 | | |
|  |  |  |  | |  | | |
| Case-mix adjustment: maternal age and ethnic background, IMD quintile, body mass index, smoking status, pre-existing hypertension, pregnancy-induced hypertension, pre-eclampsia, gestational diabetes, diabetes, obstetric cholestasis. | | | | | | |  |
| Unit characteristics: unit delivery volume and neonatal until type | | | | | | |  |

**Supplementary Table** **S4** Association between Maternity Service Restricted Ratings and Outcomes

|  | | **Restricted Maternity Service Rating** | | | |  | | |
| --- | --- | --- | --- | --- | --- | --- | --- | --- |
|  | **Outstanding** | | **Good** | | **RI/Inadequate** |  | | |
|  | IRR (95% CI) | |  | | IRR (95% CI) | | P-value | |
| **Maternal outcome** |  | |  | |  | |  | |
| EMMOI (n=398 201) | n = 16 699 | | n = 286 001 | | n = 95 501 | |  | |
| Unadjusted | 1.01 (0.65-1.57) | | ref | | 0.91 (0.74-1.11) | | 0.64 | |
| Adjusted case-mix & unit char | 0.96 (0.63-1.46) | | ref | | 0.97 (0.80-1.18) | | 0.95 | |
|  |  | |  | |  | |  | |
| OASI (n=283 826) | n = 11 319 | | n = 203 560 | | n = 68 947 | |  | |
| Unadjusted | 1.02 (0.80-1.30) | | ref | | 0.98 (0.87-1.09) | | 0.88 | |
| Adjusted case-mix & unit char | 0.88 (0.70-1.11) | | ref | | 1.05 (0.94-1.16) | | 0.35 | |
|  |  | |  | |  | |  | |
| **Neonatal outcome** |  | |  | |  | |  | |
| EPNM (n=383 387) | n = 15 724 | | n = 273 922 | | n = 93 741 | |  | |
| Unadjusted | 0.95 (0.56-1.60) | | ref | | 1.14 (0.91-1.43) | | 0.51 | |
| Adjusted case-mix & unit char | 0.95 (0.58-1.58) | | ref | | 1.19 (0.96-1.48) | | 0.26 | |
|  |  | |  | |  | |  | |
| ENAOI (n=383 033) | n = 15 710 | | n = 273 689 | | n = 93 634 | |  | |
| Unadjusted | 1.07 (0.69-1.65) | | ref | | 0.90 (0.73-1.09) | | 0.51 | |
| Adjusted case-mix & unit char | 1.07 (0.70-1.63) | | ref | | 0.94 (0.77-1.14) | | 0.75 | |
|  |  | |  | |  | |  | |
| Apgar <7 at 5 minutes (n=364 644) | n = 14 056 | | n = 263 736 | | n = 86 852 | |  | |
| Unadjusted | 0.79 (0.54-1.17) | | ref | | 1.00 (0.84-1.19) | | 0.50 | |
| Adjusted case-mix & unit char | 0.80 (0.55-1.18) | | ref | | 1.01 (0.85-1.20) | | 0.51 | |
|  |  | |  | |  | |  | |
| **Process measure** |  | |  | |  | |  | |
| Non-spontaneous birth (n= 340 655) | n = 13 618 | | n = 247 973 | | n = 79 074 | |  | |
| Unadjusted | 0.95 (0.82-1.09) | | ref | | 1.00 (0.94-1.06) | | 0.74 | |
| Adjusted case-mix & unit char | 0.96 (0.84-1.10) | | ref | | 0.98 (0.93-1.04) | | 0.71 | |
|  |  | |  | |  | |  | |
| Intrapartum caesarean (n= 342 599) | n = 14 290 | | n = 246 160 | | n = 82 149 | |  | |
| Unadjusted | 1.23 (1.04-1.45) | | ref | | 0.95 (0.88-1.02) | | **0.01** | |
| Adjusted case-mix & unit char | 1.10 (0.96-1.26) | | ref | | 0.99 (0.93-1.06) | | 0.36 | |
|  | |  | |  |  |  | | |
| Case-mix adjustment: maternal age and ethnic background, IMD quintile, body mass index, smoking status, pre-existing hypertension, pregnancy-induced hypertension, pre-eclampsia, gestational diabetes, diabetes, obstetric cholestasis. | | | | | | | |  |
| Unit characteristics: unit delivery volume and neonatal until type | | | | | | | |  |
